# Benefits and Challenges of ECHO Innovation to Reach New Interprofessional Team Members and Meet Workforce Needs

**DOI:** 10.1093/geroni/igaf122.749

**Published:** 2025-12-31

**Authors:** Lauren Gleason, Monica Long, Katherine Thompson

**Affiliations:** UChicago Medicine, Chicago, Illinois, United States; University of Chicago Medicine/SHARE Network, Glenwood, Illinois, United States; UChicago Medicine, Chicago, Illinois, United States

## Abstract

The University of Chicago GWEP has a long history of utilizing the Project ECHO model to train interprofessional teams of healthcare workers. One group not historically included in the ECHO training model is certified nursing assistants (CNAs) working in skilled nursing facilities (SNFs). This workforce is particularly vulnerable to workplace injury, burnout, and high job turnover. Using a needs assessment of CNAs to guide our program development, we created an innovative ECHO curriculum to train CNAs in SNFs, focusing on such high yield topics as empowerment, leadership, peer support, and quality improvement. Data from mixed methods assessment of barriers and facilitators to participation and learning were collected for future program quality improvement. Barriers included irregularity of participant availability due to work schedules and lack of comfort and familiarity with case-style presentations. Facilitators included ability to feel empowered in the workplace by developing additional skills and a peer community of practice. Adaptations for quality improvement included providing sample cases for discussion and tailoring topics to evolving needs of participants. Using the ECHO model to develop innovative programs may be a scalable way to meet interprofessional workforce needs as well as support vulnerable workers by developing a sense of empowerment and a community of practice. Traditional ECHO teaching strategies (e.g., case presentations) may need to be adapted based on comfort level and familiarity of the learner group.

